# MUC16 promotes triple-negative breast cancer lung metastasis by modulating RNA-binding protein ELAVL1/HUR

**DOI:** 10.1186/s13058-023-01630-7

**Published:** 2023-03-14

**Authors:** Sanjib Chaudhary, Muthamil Iniyan Appadurai, Shailendra Kumar Maurya, Palanisamy Nallasamy, Saravanakumar Marimuthu, Ashu Shah, Pranita Atri, Chirravuri Venkata Ramakanth, Subodh M. Lele, Parthasarathy Seshacharyulu, Moorthy P. Ponnusamy, Mohd W. Nasser, Apar Kishor Ganti, Surinder K. Batra, Imayavaramban Lakshmanan

**Affiliations:** 1 Department of Biochemistry and Molecular Biology, University of Nebraska Medical Center, Omaha, NE 68198-5870 USA; 2 Department of Pathology and Microbiology, University of Nebraska Medical Center, Omaha, NE 68198-5900 USA; 3 Eppley Institute for Research in Cancer and Allied Diseases, University of Nebraska Medical Center, Omaha, NE 68198-5900 USA; 4 Division of Oncology-Hematology, Department of Internal Medicine, VA Nebraska Western Iowa Health Care System, University of Nebraska Medical Center, Omaha, NE 68105-1850 USA; 5 Fred and Pamela Buffett Cancer Center, University of Nebraska Medical Center, Omaha, NE 68198-5870 USA

**Keywords:** UC16, ELAVL1/HuR, cMyc, YBX-1, YB1, MMP1, MMP3, TNBC, Metastasis

## Abstract

**Background:**

Triple-negative breast cancer (TNBC) is highly aggressive with an increased metastatic incidence compared to other breast cancer subtypes. However, due to the absence of clinically reliable biomarkers and targeted therapy in TNBC, outcomes are suboptimal. Hence, there is an urgent need to understand biological mechanisms that lead to identifying novel therapeutic targets for managing metastatic TNBC.

**Methods:**

The clinical significance of MUC16 and ELAVL1 or Hu antigen R (HuR) was examined using breast cancer TCGA data. Microarray was performed on MUC16 knockdown and scramble TNBC cells and MUC16-associated genes were identified using RNA immunoprecipitation and metastatic cDNA array. Metastatic properties of MUC16 were evaluated using tail vein experiment. MUC16 and HuR downstream pathways were confirmed by ectopic overexpression of MUC16-carboxyl-terminal (MUC16-Cter), HuR and cMyc as well as HuR inhibitors (MS-444 and CMLD-2) in TNBC cells.

**Results:**

MUC16 was highly expressed in TNBC and correlated with its target HuR. Depletion of MUC16 showed decreased invasion, migration, and colony formation abilities of human and mouse TNBC cells. Mice injected with MUC16 depleted cells were less likely to develop lung metastasis (*P* = 0.001). Notably, MUC16 and HuR were highly expressed in the lung tropic TNBC cells and lung metastases. Mechanistically, we identified cMyc as a HuR target in TNBC using RNA immunoprecipitation and metastatic cDNA array. Furthermore, MUC16 knockdown and pharmacological inhibition of HuR (MS-444 and CMLD-2) in TNBC cells showed a reduction in cMyc expression. MUC16-Cter or HuR overexpression models indicated MUC16/HuR/cMyc axis in TNBC cell migration.

**Conclusions:**

Our study identified MUC16 as a TNBC lung metastasis promoter that acts through HuR/cMyc axis. This study will form the basis of future studies to evaluate the targeting of both MUC16 and HuR in TNBC patients.

**Supplementary Information:**

The online version contains supplementary material available at 10.1186/s13058-023-01630-7.

## Background

Breast cancer is the most common malignancy and the second leading cause of cancer-related deaths in women [1]. In the USA, about 300,590 new cases and nearly 43,700 patients are expected to die in the year 2022 [1]. Basal or triple-negative breast cancer (TNBC) is a highly aggressive subtype of breast cancer with a median overall survival of approximately one year and is often associated with the development of metastases [2–4]. Therefore, identifying better prognostic markers and mechanisms associated with the development of metastasis could help develop targeted therapies and better clinical outcomes in TNBC.

Cancer antigen 125/Mucin 16 (CA125/MUC16) has been associated with cancer progression and metastasis in various cancer types, including breast cancer [5–8]. Increased MUC16 expression in breast cancer also promotes cell cycle progression (G2-M) and cell survival via JAK2/STAT3 signaling pathway [9]. MUC16 is also used as a biomarker to monitor the disease progression, recurrence, and chemotherapeutic response in ovarian cancer [10–12]. Apart from the tumorigenic role of MUC16 in breast cancer [13–15], it was observed as one of the most frequently mutated gene in metastatic breast tumors, followed by *TP53* [16, 17].

Embryonic lethal, abnormal vision, Drosophila-like protein 1 (ELAVL1), also known as Hu antigen R (HuR), is an RNA-binding protein that contains an RNA recognition motifs [18] which regulates the stability of various target mRNAs [19]*.* Elevated ELAVL1 (hereon will be referred to as HuR) expression has been observed in several cancers and regulates the stability of many cancer-associated transcripts during cancer progression and metastasis [20–23]. The function of HuR is dependent on the localization of HuR; for instance, HuR mediates its oncogenic role when it translocates from the nucleus to the cytoplasm [24]. Several small-molecule inhibitors have been developed for targeting HuR [25, 26]. MS-444 is an inhibitor that targets HuR by blocking its dimerization in the nucleus, thus preventing its cytoplasmic trafficking [26–28]. Another inhibitor, CMLD-2, disrupts the interaction between HuR and target mRNAs [25]. In breast cancer, HuR is upregulated in the cytoplasm and is correlated with poor clinical outcomes [23, 29, 30]. Y-Box-binding protein 1 (YBX-1) is an interaction partner of HUR, which is also overexpressed in many cancers and plays an important role in various cellular functions [31]. The transcription factor cMyc is overexpressed in TNBC and regulates various oncogenes and thus promotes proliferation and metastasis as well as many other cellular processess [32–34].

Recent studies have shown that cancer cells preferentially spread and metastasize to distant organs under the influence of selective cellular and molecular programs [35–37]. Numerous studies have focused on identifying cell-intrinsic determinants for such distinct organotropism during metastasis, including transcription factors, kinases, and cell surface receptors expressed on tumor cells that facilitate such preferential tropism [37–39]. However, efficient colonization in secondary organs also depends on various oncogenic proteins, which modulate the extensive survival signals and colonization activities. In this study, we evaluated the role of an oncogenic glycoprotein, MUC16 in TNBC metastasis.

## Methods

### Cell culture and stable knockdown cell line generation

TNBC cell lines: MDA MB 231, HCC1806, and HCC1937 cells, were purchased from the American Type Culture Collection (Manassas, VA, USA) and maintained in RPMI1640 medium supplemented with 10% FBS and 1X penicillin and streptomycin. Mouse basal type cell line, 4T1, was cultured in DMEM with the above-mentioned supplements. Scramble control and pSUPER-Retro-shMUC16 were transfected into phoenix cells using Lipofectamine 2000 to generate viral particles (Invitrogen, Carlsbad, CA, USA) [9, 40]. After 48 h, the supernatant (viral particles) was collected, centrifuged, filtered, and used to infect the MDA MB 231 and HCC1806 cells. Similarly, mouse-specific pSUPER-Retro-shMuc16 and scramble-shRNA were used in the 4T1 cells. The pooled population of MUC16 knockdown cells was obtained using the antibiotic selection (puromycin 4 μg/ml) and was further expanded to the confluent levels to obtain stably transfected cells. Then, MUC16 knockdown and scramble cells were used for further analyses, including functional studies. In addition, MUC16 knockdown and scramble cells were used for microarray analysis with Affymetrix GeneChip system.

### Tissue microarray, immunohistochemistry, and immunofluorescence

Immunohistochemistry (IHC) was performed in the commercially available tissue microarray (TMA)—BR10011a (US Biomax) and A202VI (Accumax), which included 185 cases and normal breast tissues (*N* = 5). Briefly, the TMAs or slides were baked overnight at 56 °C to remove the excess paraffin and hydrated in the graded alcohol (100–20%, 5 min each). Antigen was retrieved in the 0.01 M citrate buffer (0.01% Tween20) for 15 min in the microwave, and endogenous peroxidase activity was quenched by 0.3% H_2_O_2_ (1 h/dark) followed by blocking with 2.5% horse serum (Impress reagent kit, Vector Laboratories) at room temperature for 1 h. The slides were incubated overnight with MUC16 (M11 clone, Dako, dilution 1:750) and HuR (Abcam, dilution 1:500) at 4 °C, washed with PBS-Tween 20 (PBST), and further incubated with the ImmPRESS Universal anti-mouse IgG/anti-rabbit IgG for 30 min at room temperature. The brown coloration was developed by using DAB, counterstained with hematoxylin, followed by dehydration with graded alcohol (20 to 100%), air-dried, and mounted with PerMount [40]. MUC16 immunostaining was evaluated by a trained pathologist, blinded to the clinical information. The xenograft tissues were quantitatively assessed by Fiji-Image J software [41].

For immunofluorescence studies, the tissues were processed according to the above-mentioned process till the antigen retrieval process. The tissues were then blocked with 10% normal goat serum (Jackson Immunoresearch Labs, Inc., West Grove, PA, USA) for 30 min followed by incubation with primary antibodies: MUC16 (Mouse, 1:750) and HuR (Rabbit, 1:500) for overnight at 4 °C. Next day, the cells were washed with PBS (5 min, 3×) and then incubated with fluorescein isothiocyanate-conjugated anti-mouse and Texas red-conjugated anti-rabbit secondary antibodies (Jackson Immunoresearch Labs, Inc.) for 30 min at room temperature in the dark. Further the tissues were washed with PBS (5 min, 3×) with gentle shaking and finally mounted with an anti-fade vectashield mounting medium containing DAPI (Vector Laboratories, Burlingame, CA, USA). Images were acquired with the LSM710 microscope (Carl Zeiss GmbH, Jena, Germany).

### Quantitative real-time PCR

Quantitative real-time PCR was performed as previously described [40]. Total RNA was isolated using the Qiagen Kit (Germantown, MD, USA). Approximately two micrograms of total RNA were used for the cDNA synthesis using reverse transcriptase SuperScript^®^II (Invitrogen, Carlsbad, CA, USA). Quantitative PCR was performed using the SYBER Green. The difference between the test and control was depicted as a fold change.

### Immunoblot

Total protein was isolated using the RIPA buffer (50 mM Tris–HCl, 150 mM NaCl, 1% NP-40, 0.5% sodium deoxycholate, and 0.1% SDS) containing 1X protease inhibitor cocktail. About 20–40 μg of total protein was run in 10% SDS-PAGE gel for the signaling studies, while the MUC16 protein was resolved in the 2% SDS-agarose gel. The proteins were transferred to the PVDF membrane, blocked with 5% skimmed milk, and probed with the respective primary antibodies-MUC16 (M11 clone, Mouse 1: 1000, Dako), HuR (Rabbit, 1:1000, Abcam), cMyc (Mouse 1:1000, Santa Cruz Biotechnology), YBX-1 (Rabbit, 1:1000, Cell Signaling), Vimentin (Rabbit, 1:1000, Cell signaling) Zeb1 (Rabbit, 1:1000, Cell signaling), HA tag (Rabbit, 1:1000, Cell Signaling), and β-actin (1:2000, Sigma-Aldrich) for overnight at 4 °C. The membranes were then washed (3×, 10 min) in PBST at room temperature, probed with the appropriate secondary antibodies (1:5000 dilutions) for 1 h, and washed (3×, 10 min) with PBST. The signal was detected with the ECL chemiluminescence kit (Amersham Bioscience, Buckinghamshire, UK).

### Cell invasion, motility, colony formation, and scratch assays

For the invasion, and migration assays, about 1 million of scramble and MUC16 knockdown cancer cells (MDA MB 231, HCC1806, and 4T1) were seeded (serum-free condition) in the 8 µm pore size six-well inserts (Becton Dickinson, Franklin Lakes, NJ, USA), while 10–20% serum containing medium was used as a chemoattractant in the bottom chamber. After 24 h, the cancer cells that are migrated to the lower chamber were stained with the Quick-Diff kit staining solution and then counted at eight different random fields. The average number of migrated cells per representative field was plotted using the GraphPad software. Colony formation experiment was performed as described previously [42]. We performed scratch assay in MUC16-Cter and HuR overexpressed cells and respective control cells using our lab protocol [40].

### Tail vein injection

Approximately 1 million viable RFP-labeled MDA MB 231 (Scramble and shMUC16) in 50 µl PBS was injected via the tail vein of nude mice. The mice were monitored every week for metastasis by IVIS imaging. After 30 days, the mice were killed, and tissues were collected for further investigation. The mouse studies were performed in accordance with the US Public Health Service ‘Guidelines for the Care and Use of Laboratory Animals’ under an approved protocol by the Institutional Animal Care and Use Committee, University of Nebraska Medical Center.

### RNA immunoprecipitation (RIP) and PCR array

RIP was performed using an HuR antibody [43, 44]. The HCC1806-scramble and HCC1806-shMUC16 cells were cultured and lysed using the polysome lysis buffer (1000 mM KCl, 50 mM MgCl_2_, 100 mM HEPES–NaOH pH 7, 5% NP-40) supplemented with RNase and 1× protease inhibitors. The lysates were pre-cleared by adding 1 μg of IgG1 (BD Bioscience) and 50 μl of Protein-A/G Sepharose beads swollen in the NT2 buffer with 5% BSA. The beads were coated by adding either IgG1 (BD Biosciences, San Diego, CA) as control or anti-HuR antibody and incubated overnight at 4 °C. After extensive washes of pre-coated Protein-A/G Sepharose beads, the pre-cleared lysate was added and incubated for 4 h at 4 °C, and then, 30 μg of proteinase K was added to digest protein by incubation at 55 °C for 30 min. The RNA-IP’ed samples were then reverse transcribed into cDNA and used to perform the cDNA array (PAHS-028Z, QIAGEN) to identify the HuR target mRNAs.

### Overexpression of MUC16-Cter and HuR in TNBC cells

HCC1937 cells were seeded in the 60 mm petri dish and transfected with either MUC16-Cter (114 amino acids) or pSecTag2C plasmids (Invitrogen-Life Technologies) as described previously [40, 45]. The experiments were performed by transfecting the HuR (Catalog #121162, Addgene) with Lipofectamine 2000 in the MUC16 silenced HCC1806 cells (HCC1806-shMUC16). The HuR overexpressed (HCC1806-shMUC16-HuR) and vector control (HCC1806-shMUC16-Vector) cells were used for the scratch assays.

### HuR inhibitors treatment (MS-444 and CMLD-2), cell viability, and migration assays

MDA MB 231 and HCC1806 cells were seeded at the sub-confluent levels in 96-well plates and treated with the increasing concentrations of MS-444 and CMLD-2 for 48 h at 37 °C [25, 26]. The cell viability of MS-444 and CMLD-2 was determined using the MTT assays [40]. Based on the IC_50_ value of MS-444 and CMLD-2, MDA MB 231 and HCC1806 cells were treated for 48 h, and lysates were collected for the western blot analyses. For migration experiments, the 50,000 cells (MDA MB 231 and HCC1806) were seeded in the 12-well inserts (8 μm pore size) and about 10 μM drug treatment (MS-444) was done. After 24 h treatment, the inserts were processed as mentioned earlier in ‘Cell Invasion, motility, colony formation, and scratch assays’ section.

### Bioinformatic analysis

The expression profile of the MUC16 was performed at the default settings of the software (https://kmplot.com/analysis/). The MUC16 protein probe-Q8WXI7 and HuR protein probe-Q15717 were used to estimate the survival outcome for the patients with high and low MUC16 expression in breast cancer [46]. TCGA-METABRIC dataset was used for MUC16 and HuR expression in different subtypes and correlations.

### Data analysis and statistics

All experiments were performed in triplicates. Results were expressed as means ± SD. Statistical significance was evaluated with the Student’s *t*-test using GraphPad Prism 8.1.2 software. *P* < 0.05 was considered statistically significant.

## Results

### MUC16 expression and poor survival outcomes in breast cancer

**Fig. 1:**
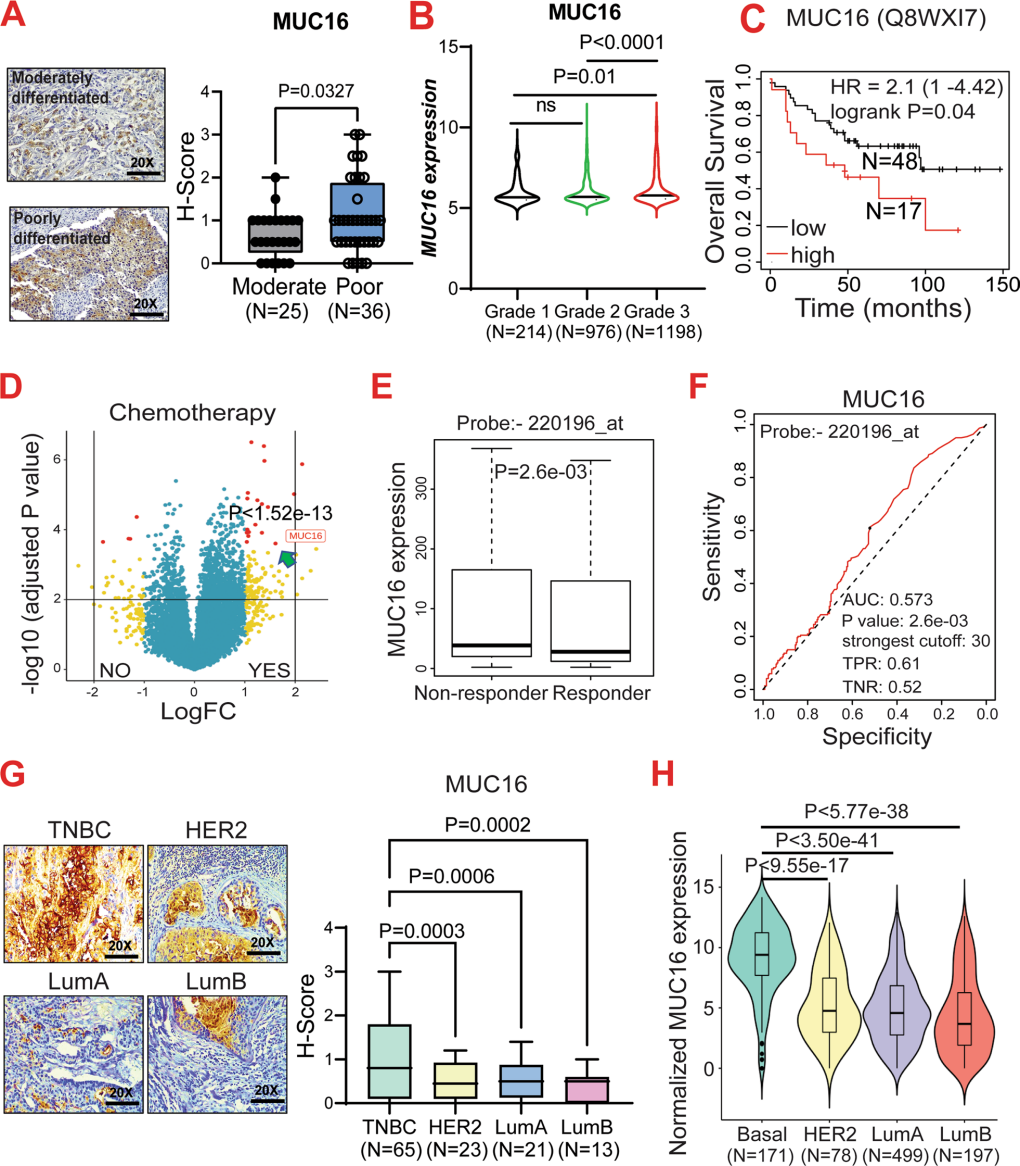
Overexpression of MUC16 in TNBC patients. **A.** Expression of MUC16 is highly upregulated in the poorly differentiated patients (*N* = 36) compared to moderately differentiated samples (*N* = 25). **B.** In silico TCGA-BRCA analysis indicating grade-wise expression of MUC16 transcript. **C.** Kaplan–Meier survival plot (https://kmplot.com) showing significant reduction in overall survival of breast cancer patients with high MUC16 (Q8WXI7) protein expression (220,196 probe was used to identify the MUC16 gene) **D**. Volcano plot of TCGA-BRCA dataset indicating high levels of MUC16 mRNA in breast cancer patients that have undergone chemotherapy compared to chemotherapy-naive patients. **E**. Box plot showing increased MUC16 expression in non-responders (any chemotherapy) compared to responders in breast cancer. **F**. AUC curve of MUC16 (220196_at) based on the 5-year relapse-free survival of breast cancer after any chemotherapy (http://www.rocplot.org). **G.** Box plots of IHC scores showing upregulation of MUC16 expression in TNBC patients compared to other breast cancer subtypes. **H.** Similarly, in silico dataset (TCGA-METABRIC) indicated upregulation of MUC16 in TNBC patients (*N* = 171) compared to HER2 (*N* = 78), Lum A (*N* = 499), and Lum B (*N* = 197)

To assess the clinical impact of MUC16 in breast cancer, we performed immunohistochemistry (IHC) in a breast cancer TMA, which consisted of moderately differentiated (*N* = 25) and poorly differentiated (*N* = 36) tumors. We observed that MUC16 expression was significantly higher in poorly differentiated tumors (*P* = 0.0327) compared with moderately differentiated tumors (Fig. 1A). Analysis of the BRCA METABRIC cohort also indicated a significant increase in the MUC16 expression in the more undifferentiated tumors (G3 versus G1, *P* = 0.0173, G3 versus G2, *P* < 0.0001) (Fig. 1B). Furthermore, Kaplan–Meier survival curve analysis in the Tang 2018 breast cancer cohort showed that high MUC16 (Probe: Q8WXI7) protein expression (*N* = 17) was associated with significant (*P* = 0.046) reduction in the overall survival compared to patients with low MUC16 expression (*N* = 48) [46], suggesting a possible role for MUC16 in breast cancer pathogenesis and survival (Fig. 1C). To understand the potential role of MUC16 in response to chemotherapy in breast cancer patients, we analyzed the expression of MUC16 in the TCGA-BRCA cohort in the therapy naive patients and those treated with any chemotherapeutic regimen. We found *MUC16* mRNA was one of the top upregulated gene (*P* < 1.52e−13) in response to therapy compared to treatment naive patients (Fig. 1D). More importantly, there was a significant upregulation of MUC16 expression in the non-responder breast cancer patients compared to the chemotherapy responders, with an AUC (sensitivity and specificity) of 0.573 (*P* = 2.6e−03) (Fig. 1E–F). These findings indicated that MUC16 may be associated with breast cancer aggressiveness, survival, and chemotherapy resistance.

### MUC16 is highly expressed in the TNBC subtype

Since breast cancer is highly heterogeneous at the molecular level, we analyzed the MUC16 expression in the different molecular subtypes of breast cancer. Our previous data have shown no expression of MUC16 in healthy breast tissues [9]. We observed a robust increase in MUC16 expression in the TNBC tumor tissues (*N* = 65) compared with other subtypes-HER2 positive (*N* = 23, *P* = 0*.*0003), luminal A (*N* = 21, *P* = 0*.*0006), luminal B (*N* = 13, *P* = 0*.*0002), and tumors (Fig. 1G). Similarly, the TCGA-METABRIC cohort also showed a significant increase in the *MUC16* expression in the TNBC subtype (*N* = 171) compared to the other subtypes—HER2 (*N* = 78, *P *= 9.55105E−17), Luminal A (*N* = 499, *P* = 3.5034E−41), and Luminal B (*N* = 197, *P* = 5.77798E−38) (Fig. 1H). Overall, these findings indicate that MUC16 is overexpressed in the TNBC and may be associated with the poor outcomes seen in this subtype.

### MUC16 is associated with RNA bio-synthetic and metastasis pathways in TNBC

**Fig. 2:**
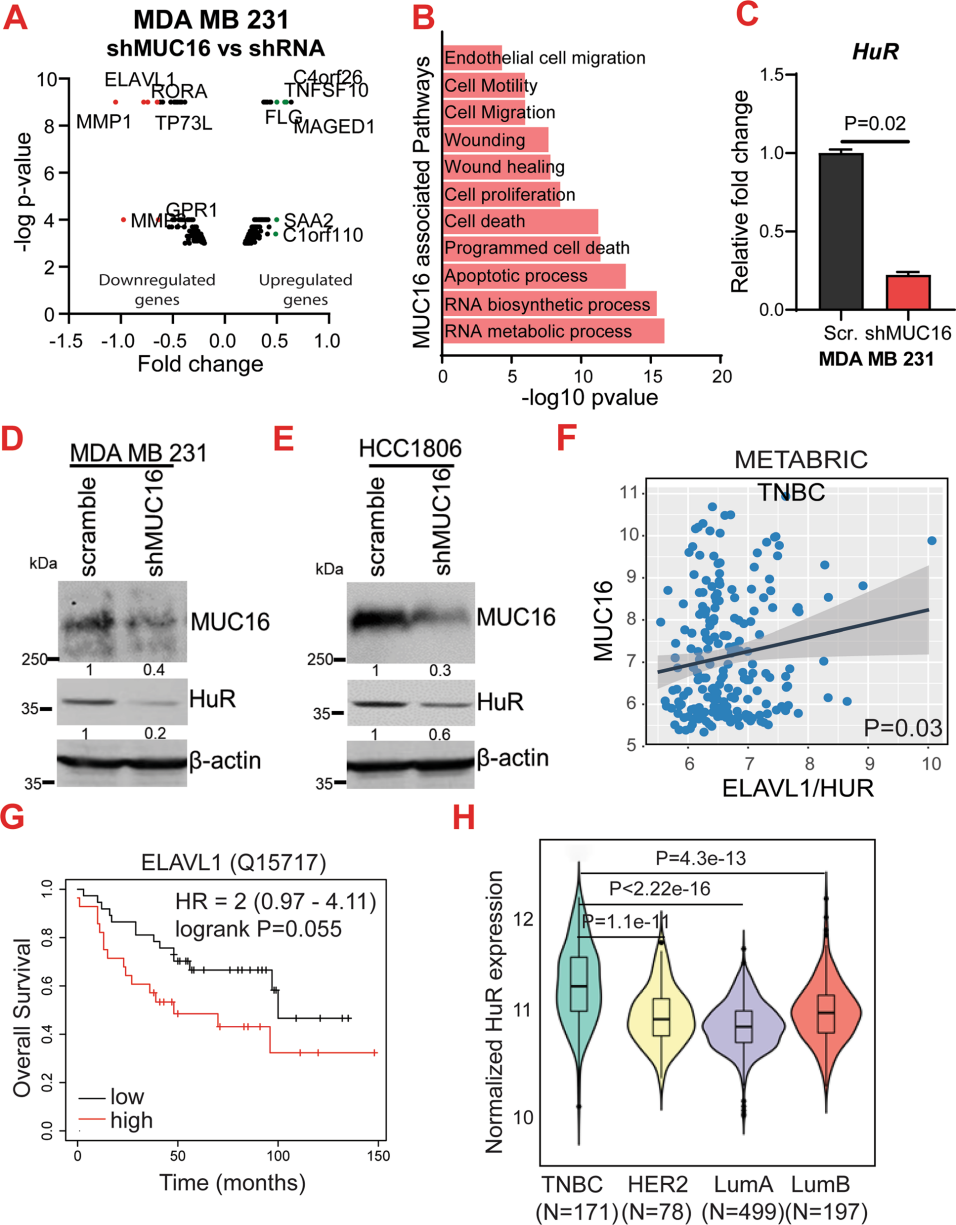
MUC16 regulates HuR in TNBC cells. **A**. Volcano plot demonstrating decreased expression of ELAVL1/HuR, MMP1, and MMP3 in MUC16 knockdown TNBC cells. **B**. Gene ontology analysis of MUC16-associated pathways suggests its role in RNA biosynthesis, apoptotic and cell motility, and migration pathways. **C**. Bar diagram of qRT-PCR showing decreased HuR expression in MUC16 knockdown cells (MDA MB 231) compared to scramble. Actin was used as an internal control**. D**, **E**. Western blot results indicated that HuR was downregulated in MUC16 knockdown (MDA MB 231 and HCC1806). **F**. METABRIC dataset indicated co-expression of MUC16 and HuR in TNBC. **G**. Kaplan–Meier survival plot (Q15717) showing HuR is associated with poor survival in breast cancer patients (https://kmplot.com). **H**. HuR expression in different breast cancer subtypes (TCGA-METABRIC)

To investigate the MUC16-associated molecular mechanism(s) and pathways in the pathogenesis of TNBC, we performed a gene expression analysis in the MUC16 knockdown MDA MB 231 (MDA MB 231-shMUC16) and corresponding scramble (MDA MB 231-shRNA) cells. Our transcriptome analysis (microarray) indicated a significant downregulation of ELAVL1 or HuR, *MMP1*, and *MMP3* genes in the MUC16 knockdown cells (Fig. 2A). Next, we performed the pathway analysis (Gene ontology) using the top-downregulated genes following MUC16 knockdown (MDA MB 231-shMUC16). We noticed that RNA biosynthesis, RNA metabolism, apoptosis, and regulation of programmed cell death, cell proliferation, and metastasis pathways were significantly associated with MUC16 (Fig. 2B). Further analysis of the transcriptomic data by qRT-PCR also confirmed significant downregulation of *HuR, MMP1*, and *MMP3* in the MUC16 knockdown cells (Fig. 2C, Additional file 1: Fig. S1A–C). Western blot analysis also indicated that expression of HuR was drastically decreased in MUC16 knockdown TNBC MDA MB 231 and HCC1806 cells (Fig. 2D–E). Furthermore, we observed that MUC16 was significantly co-expressed with HuR in TNBC patient samples (Fig. 2F). Like MUC16, patients with high HuR protein expression (Q15717) had a significantly worse overall survival compared to those with low HuR expression breast cancer patients (*P* = 0.055) [46] (Fig. 2G). Furthermore, HuR was also significantly overexpressed (TCGA-METABRIC) in the TNBC subtype as compared to other subtypes (Fig. 2H). Though MMP3 and MMP1 are downregulated in MUC16 knockdown cells, the expression of MMP3 and MMP1 was not elevated in TNBC (Additional file 1: Fig. S1D–G). In addition, the expression of MMP3 and MMP1 was significantly high in HER2 enriched tumors as compared to other subtypes (TNBC or basal type) (Additional file 1: Fig. S1F, G). Hence, we selected HuR to understand the MUC16/HuR role in TNBC.

### Effect of MUC16 on invasion, migration, and colony formation abilities of human and mouse TNBC cells

**Fig. 3:**
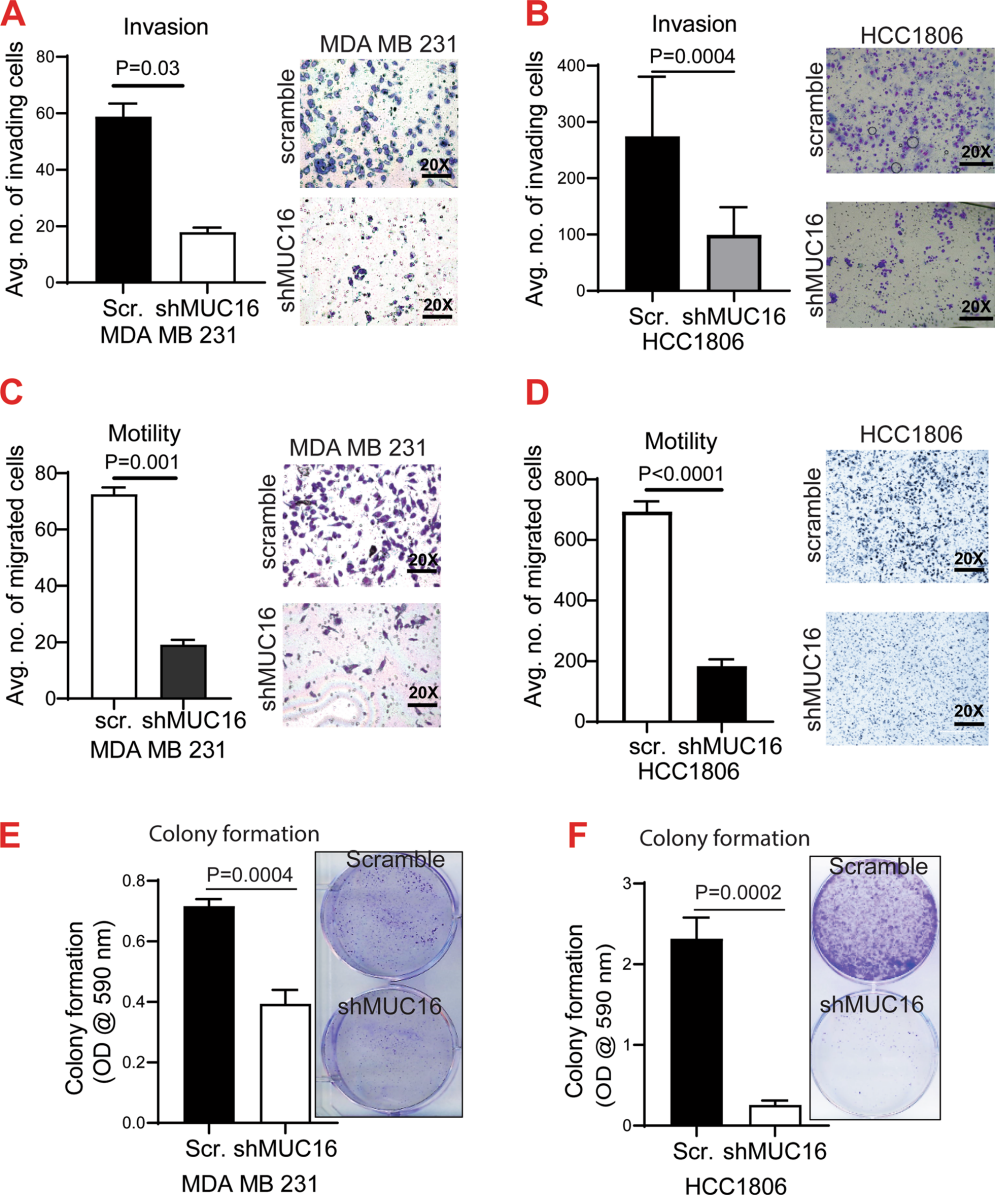
Effect of MUC16 on breast cancer cell invasion and motility and colony formation. **A**, **B**. Matrigel-coated Boyden chamber transwell assay showed decreased invasion upon MUC16 knockdown cells compared to control cells. **C**, **D**. Migration capacity of MUC16 knockdown cells (MDA MB 231-shMUC16 and HCC1806-shMUC16). **E**, **F**. Colony formation ability of MUC16 knockdown cells (MDA MB 231-shMUC16 and HCC1806-shMUC16)

To determine the functional role of MUC16 in TNBC metastasis, in vitro*,* MDA MB 231-shMUC16 and HCC1806-shMUC16 and respective scramble cells were seeded in the Matrigel-coated Boyden chamber transwell inserts for 24 h. MUC16 knockdown cells (MDA MB 231-shMUC16 and HCC1806-shMUC16) showed a significant decrease in invasion (*P* = 0.03 and *P* = 0.0004) (Fig. 3A–B), migration (*P* = 0.001 and *P* < 0.0001) (Fig. 3C–D), and colony formation (*P* = 0.0004 and *P* = 0.0002) abilities (Fig. 3E–F) compared to the scramble controls. There was decreased expression of mesenchymal marker Vimentin in MUC16 knockdown cells (Additional file 2: Fig. S2A and B). Additionally, Muc16 knockdown in mouse basal or TNBC cells 4T1 (4T1-shMuc16) showed decreased *Muc16* (Additional file 2: Fig. S2C), and *HuR* expression compared to scrambled control (Additional file 2: Fig. S2D). Further, Muc16 knockdown cells (4T1-shMuc16) also showed a significant reduction in invasion, motility, and colony formation properties compared to control (Additional file 2: Fig. S2E–G). The mesenchymal marker Zeb1 was decreased in Muc16 knockdown (4T1-shMuc16) cells compared to scramble control cells (Additional file 2: Fig. S2H). Overall, these findings suggest that MUC16 may be involved in TNBC metastasis.

### MUC16 is associated with lung metastasis of TNBC

**Fig. 4:**
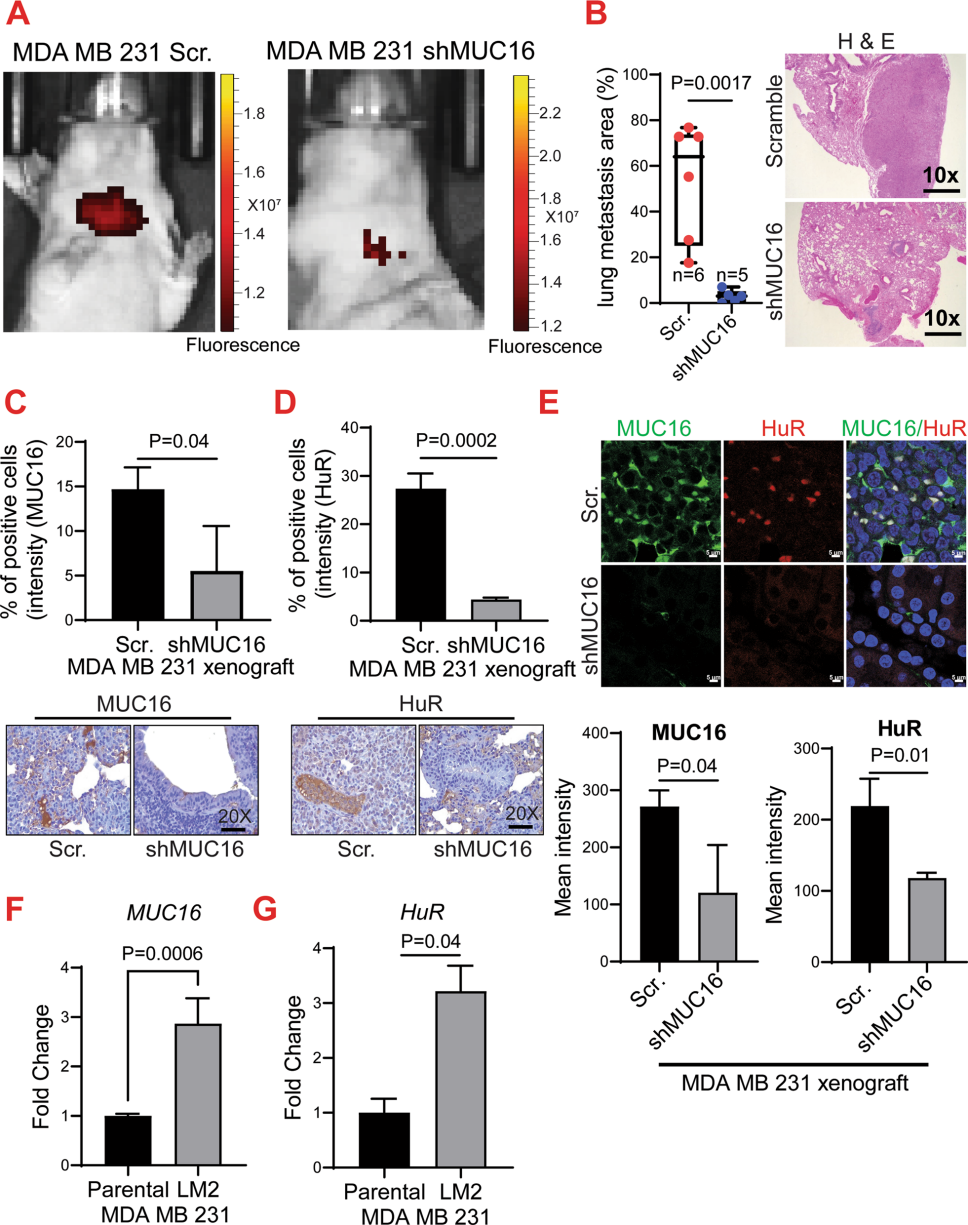
Effect of MUC16 knockdown on TNBC lung metastasis. **A**. Representative IVIS image of scramble and MUC16 knockdown cells injected mice. **B**. Bar diagram and histological images were showing decreased percentage area of lung metastasis in nude mice injected with MUC16 knockdown (*N* = 5) as compared to scramble controls (*N* = 6). **C**, **D**. IHC images and corresponding quantification represent that decreased expression of MUC16 and HuR in MUC16 knockdown lung xenograft compared to control (20× magnification). **E**. Confocal image indicating decreased MUC16 and HuR expression in the MUC16 knockdown cells injected xenograft as compared to scramble control xenografts. **F**, **G**. qRT-PCR data showing upregulation of MUC16, and HuR in the MDA MB 231 lung tropic cells (LM2) as compared to parental MDA MB 231 cells

To prove the role of MUC16 in TNBC metastasis, we performed a tail vein injection experiment using MUC16 knockdown and scramble control cells (1 × 10^6^ RFP-labeled cells/mouse) in athymic nude mice. The metastases in the athymic nude mice were routinely monitored by IVIS imaging (Fig. 4A). We observed that mice injected with MUC16 knockdown cells (*N* = 5) developed less lung metastasis as compared to mice injected with scramble control (*N* = 6) cells. Histological analysis (H&E) of the lung tissues also showed a reduction in metastases with the presence of micrometastases in MUC16 knockdown cells injected lung xenograft (*P* = 0.0017), as compared to macrometastasis in the scramble control cells injected xenografts (Fig. 4B). We did not observe any other marked metastases incidence between MUC16 silenced and scramble cells injected mice. Next, we wanted to determine the expression of MUC16 and HuR in metastatic lung tumor xenografts. The expression of MUC16 and HuR was low in lung tumors arising from MUC16 knockdown cells compared to scramble control cells, respectively (Fig. 4C–D). We next performed a confocal analysis of MUC16 and HuR in the lung tumor xenografts and found that HuR distribution was higher in the cytoplasmic region in tumor tissues of scramble cells compared to MUC16 knockdown cells (Fig. 4E). Of note, lung tropic cells MDA MB 231-LM2 also showed elevated expression of MUC16 (Fig. 4F) and HuR (Fig. 4G) compared to parental MDA MB 231. Overall, these findings suggested that MUC16 induces TNBC lung metastasis by modulating HuR and its target genes.

### Expression of MUC16 in breast cancer lung metastatic tissues

To investigate the clinical impact of MUC16 on breast cancer metastasis, we analyzed MUC16 expression in publicly available patient datasets of metastatic breast cancer. The GSE14020 (*N* = 36) dataset which includes various sites of metastases, such as bone (*N* = 8), brain (*N* = 7), liver (*N* = 5) and lung (*N* = 16). The datasets were individually Robust Multichip Average (RMA) normalized and further z-normalized to reduce experiment or platform-induced bias. MUC16 was highly expressed in the lung, brain, and bone metastases tissues, indicating that MUC16 may have a role in metastasis (Additional file 3: Fig. S3A). Similarly, elevated levels of HuR were observed in lung and other metastatic organs (Additional file 3: Fig. S3B). Another dataset (GDC data portal) also shows the MUC16 expression is significantly upregulated in various metastases (individual metastases not available) (*N* = 40) as compared to primary breast tumors (*N* = 1334) (Additional file 3: Fig. S3C), indicating that the MUC16/HuR axis is associated with breast cancer metastasis.

### MUC16-associated HuR targets in TNBC

**Fig. 5:**
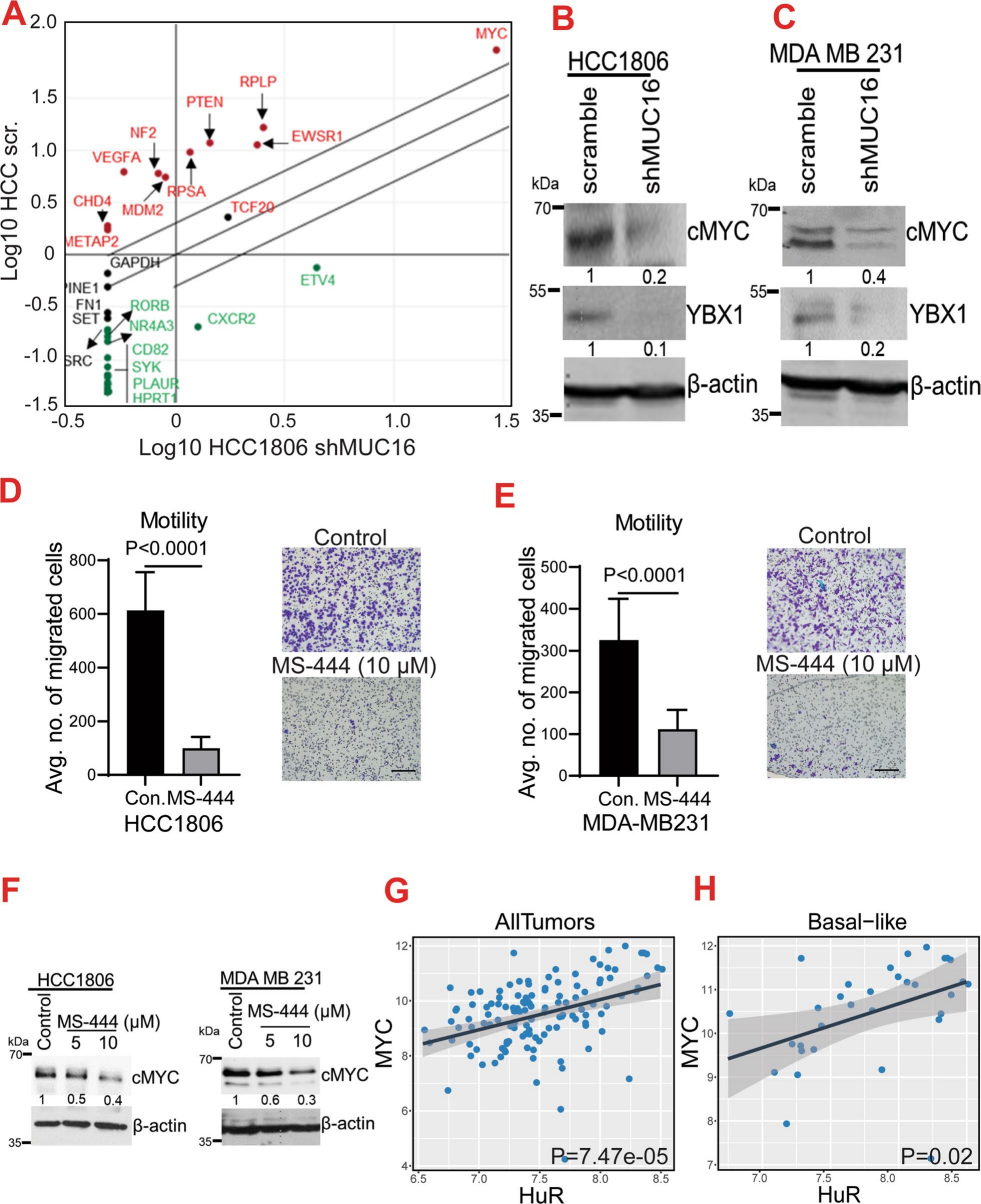
MUC16-associated HuR targets in TNBC. **A**. Scatter plot of human metastasis array after RIP assay with HuR antibody indicating the MUC16-associated HuR target genes in TNBC cells. Scatter plot showing upregulation of *Myc*, *EWSR1*, *VEGFA*, *RPLP*, *NF2*, and *CHD4* in scramble (HCC1806-SCR) cells; while *ETV4*, *CXCR2, RORB*, and *CD82* genes were downregulated in the MUC16 knockdown (HCC1806-shMUC16) cells. **B**, **C**. Western blot showing decreased levels of cMyc and YBX-1 expression upon MUC16 knockdown. **D**, **E**. Represents that MS-444 treatment and its effect on migration properties of HCC1806 and MDA MB 231 cells. **F**. Western blot showing decreased expression of cMyc upon MS-444 treatments in HCC1806 and MDA MB 231 cell lines. **G**, **H**. TCGA-METABRIC analysis indicated co-expression of HuR and Myc in breast cancer and TNBC subtype

To determine the MUC16 mediated HuR target genes in TNBC, we performed RNA immunoprecipitation (RIP) with HuR antibody, followed by a tumor metastasis PCR array. First, HuR target genes were pull-down using HuR antibody from scramble control (HCC1806-SCR) cells and MUC16 knockdown (HCC1806-shMUC16) cells. Then, immunoprecipitated HuR transcripts were used for PCR analysis (human tumor metastasis cDNA array). This showed enrichment of several genes, namely Proto-Oncogene, BHLH Transcription Factor (*Myc*), EWS RNA-Binding Protein 1 (*EWSR1*), Ribosomal Protein Lateral Stalk Subunit (*RPLP*), Phosphatase and Tensin Homolog (*PTEN*), MDM2 Proto-Oncogene (*MDM2*), and Neurofibromin 2 (*NF2*) in scramble cells. ETS Variant Transcription Factor 4 (*ETV4*), C-X-C Motif Chemokine Receptor 2 (*CXCR2*), and CD82 molecule (*CD82*) were downregulated in MUC16 knockdown cells (Fig. 5A). These molecules were validated by western blot analysis; we observed a reduction of cMyc expression in the MUC16 knockdown cells (HCC1806-shMUC16 and MDA MB 231-shMUC16) compared to scramble controls (Fig. 5B–C). These data suggest that MUC16 regulates cMyc through HuR, and these genes may be required for TNBC growth and metastasis. Of note, the HuR interaction partner YBX-1 protein was also reduced in MUC16 knockdown cells compared to scramble control cells (Fig. 5B–C), suggesting that MUC16 regulates HuR and YBX-1 during post-transcription of cMyc in TNBC cells.

### Pharmacological inhibition of HuR inhibits cMyc expression in TNBC cells

We used two pharmacological inhibitors of HuR, MS-444, and CMLD-2, to investigate the effect of HuR on cMyc expression. MS-444 blocks HuR dimerization in the nucleus and thus prevents its cytoplasmic trafficking [28], while CMLD-2, an inhibitor of HuR-ARE (adenine-uridine rich elements) interaction, binds to HuR protein and disrupts its interaction with ARE containing mRNAs [25]. We performed the cell viability assays using HuR inhibitors, MS-444 (Additional file 3: Fig. S3D–E), and CMLD-2 (Additional file 3: Fig. S3F) to identify the IC_50_ concentration. Next, we performed a transwell migration assay (24 h) to determine the effect of MS-444 on migration of TNBC cells, the results show that 10 µM concentration of MS-444 was significantly inhibits the migration of TNBC cells (Fig. 5D–E). Then, MDA MB 231 and HCC1806 cells were treated with MS-444 and CMLD-2 for 48 h. Western blot analyses showed that MS-444 inhibitor (10 µM) effectively inhibited the cMyc expression in both the cell lines, MDA MB 231 and HCC1806 (Fig. 5F). Similarly, decreased cMyc expression was observed in the CMLD-2 treated MDA MB 231 cells (Additional file 3: Fig. S3G). HuR was strongly co-expressed with cMyc in the same breast cancer patient cohort and TNBC samples (Fig. 5G–H). These findings suggest that cMyc is a direct target of HuR in TNBC cells.

### Mechanism of MUC16 mediated HuR/cMyc pathways in TNBC cells

**Fig. 6:**
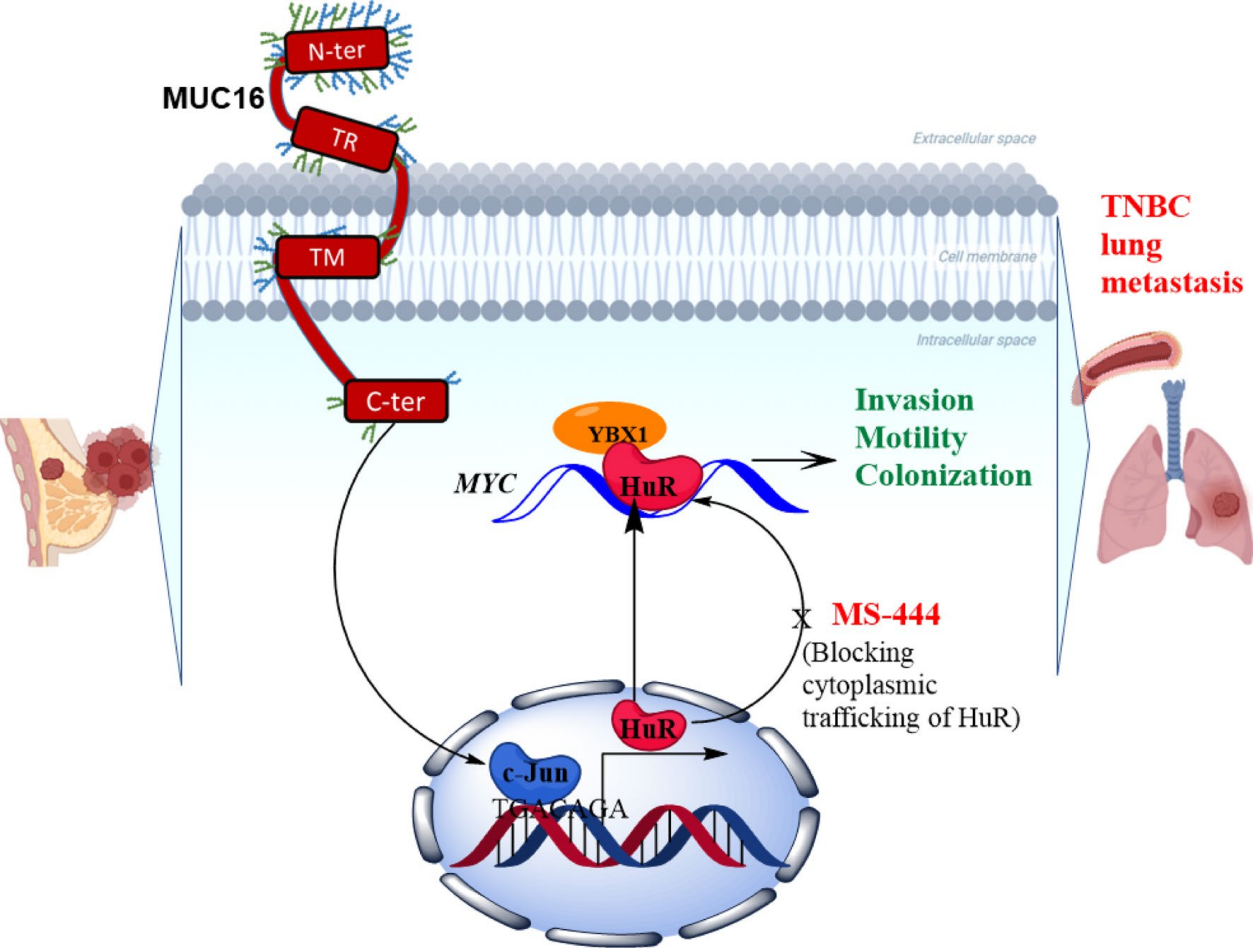
MUC16/HuR signaling in TNBC lung metastasis. The schematic diagram represents that MUC16 regulates the ELAVL1/HuR that leads to TNBC lung metastasis through cMyc signaling

To understand the mechanisms of MUC16 mediated pathways in TNBC, we ectopically overexpressed the MUC16-Cter (114aa) in HCC1937 cells. Upon overexpression of MUC16-Cter (Additional file 4: Fig. S4A), we observed the increased expression of HuR, suggesting that MUC16 regulates HuR in TNBC cells. In addition, MUC16-Cter transfected cells had significantly increased wound healing capacity as compared to vector control cells (Additional file 4: Fig. S4B). For further confirmation, we overexpressed HuR in MUC16 knockdown HCC1806 (HCC1806-shMUC16-HuR) (Additional file 4: Fig. S4C) and observed that increased expression of HuR resulted in increased expression cMyc (Additional file 4: Fig. S4C). However, the expression of HuR in MUC16 knockdown cells (HCC1806-shMUC16-HuR) did not show a significant impact on migration (Additional file 4: Fig. S4D), suggesting that the presence of MUC16 is essential for TNBC cell migration. Overall, we identified the new HuR target cMyc in TNBC that is associated with MUC16. Altogether, we observed that MUC16 regulates the HuR and its target cMyc during TNBC lung metastasis (Fig. 6).

## Discussion

TNBC subtype represents approximately 15% of all breast cancer subtypes [47]. TNBC is more likely to occur in younger women and has an abysmal prognosis [48–50]. TNBC frequently metastasizes to the lungs (36.9%), brain (25%), and bone (40%) and is associated with shorter survival [51]. The survival of metastatic TNBC patients is very poor due to therapy resistance and lack of clinically relevant biomarkers [2–4]. Our previous reports indicated that MUC16 could induce the G2-M transition of breast cancer cells by interacting with Janus kinase 2 (JAK2), which in turn enhances the phosphorylation of STAT3 (Y705) and Aurora Kinase A [9].

MUC16 is overexpressed in poorly differentiated breast tumors and overexpressed in the TNBC subtype as compared to other subtypes. MUC16 is significantly elevated in breast cancer patients who after chemotherapy. Also, we observed increased MUC16 expression in chemotherapy non-responders, suggesting a possible role for MUC16 in chemotherapy resistance.

Previous studies demonstrated the overexpression of MUC16 and its impact on migration and invasion of cancer cells [7, 45, 52]. Our in vitro metastatic experiments demonstrated that MUC16 is required for the invasion, migration, and colonization of TNBC cells. Similarly, MUC16 knockdown cells were less likely to develop lung metastasis. Further, MUC16 was significantly overexpressed in lung tropic cells, and patient data also revealed that MUC16 is elevated in breast cancer lung cancer metastatic tissues, suggesting that MUC16 is involved in breast cancer lung metastasis.

Our previous study has demonstrated that MUC16 promotes pancreatic cancer metastasis by activating the epithelial to mesenchymal phenotype through FAK activation [53]. Further, we have shown that MUC16 promotes lung cancer migration via STAT3/glucocorticoid receptor (GR)/testis-specific protein Y-encoded-like 5 (TSPYL5) axis. In the current study, we identified that MUC16 regulates RNA-binding protein HuR in TNBC and is associated with TNBC metastasis. We previously reported that MUC16 mediates c-Jun activity through STAT3 (Y705) [9]. STAT3 and c-Jun have strong binding affinities, which regulate the transcription of various oncogenes [54], suggesting that MUC16 may regulate HuR gene expression via STAT3/c-Jun. Our PROMO and TRANSFAC promotor studies indicated that c-Jun is a potential transcription factors for HuR through binding on promoter (TGACAGA, AAGGTCA, ATTGTCA, and GTTATTCTT) region of HuR gene. Our gene ontology studies indicated that MUC16 was found to be linked to RNA biosynthesis, cell death, and cell migration pathways, suggesting that MUC16 may mediate these pathways during breast cancer progression. Following that our RNA-IP and cDNA array experiment demonstrated that HuR is directly binding with Myc in TNBC cells. Further, cMyc expression was drastically decreased in MUC16 knockdown TNBC cells. In addition, we also observed that HuR interaction partner YBX-1 was drastically reduced in MUC16 knockdown cells, suggesting that MUC16 activates HuR/YBX-1 axis during post-transcriptional regulation of various oncogenes, including cMyc. Furthermore, pharmacological inhibition of HuR (MS-444 and CMLD-2) drastically reduced cMyc expression and migration abilities of TNBC cells, indicating that HuR regulates cMyc in TNBC cells. In addition, MUC16 mediated HuR pathway was confirmed by MUC16 as well as HuR expression models, the mechanistic studies clearly indicating that MUC16 is essential for HuR mediated TNBC cell migration through cMyc activation.

## Conclusions

Overall, our study has demonstrated that MUC16 is overexpressed in TNBC subtype and mediates TNBC cell invasion and lung metastasis. Mechanistically, MUC16 regulates the HuR for cMyc expression that mediates TNBC cell invasion, migration, and lung metastasis (Fig. 6). Overall, our studies in the future will aim to develop monoclonal antibodies or inhibitors targeting MUC16 along with HuR inhibitors to prevent TNBC lung metastasis and improve TNBC patient outcomes.

## Supplementary Information

**Additional file 1**: **Fig. S1**. Validation of MUC16 knockdown genes. A–C. MUC16-associated genes (MMP1 and MMP3) were validated in MDA MB 231 cells upon MUC16 knockdown. D–E. GEPIA dataset shows the expression of MMP1 and MMP3 in breast cancer patients. F, G. expression of MMP1 and MMP3 in different subtypes of breast cancer (TCGA-METABRIC).

**Additional file 2**: **Fig. S2**. MUC16 knockdown cells exhibit decreased metastatic markers. A–B. MUC16 knockdown (MDA MB 231-shMUC16 and HCC1806-shMUC16) shows decreased mesenchymal marker Vimentin. C–D. Bar diagram showing Muc16 knockdown and its impact on HuR expression in mouse TNBC 4T1 cells. E–G. Matrigel-coated Boyden chamber invasion, motility and colony formation assay indicating decreased invasion, migration and colony formation abilities in Muc16 knockdown in 4T1 cells. H. Decreased mesenchymal marker Zeb1 in Muc16 knockdown 4T1 cells. β-actin was used as an internal control.

**Additional file 3**: **Fig. S3**. Pharmacological inhibition of HuR and cMyc expression: A, B In silico analysis indicated the expression of MUC16 and HuR in various breast cancer metastatic tissues such as lung, brain, bone, and liver. Among all sites of breast cancer metastases, the level of MUC16 is high in lung metastases tissues. C. In silico data (GDC data portal) analysis indicated that MUC16 expression is significantly high in breast cancer metastatic tissues (individual metastasis not available). D–F. MTT assay shows the effect of MS-444 and CMLD-2 on the viability of HCC1806 and MDA MB 231 cells. (G) CMLD-2 is effectively inhibiting the cMyc expression in MDA MB 231 cells.

**Additional file 4**: **Fig. S4**. Overexpression of MUC16-Cter and HuR in TNBC cells: A, B Ectopic expression of MUC16-Cter in TNBC cells HCC1937 and its impact of HuR expression and significantly increased wound healing properties. C. Overexpression of HuR induced cMyc expression in MUC16 silenced HCC1806 cells (HCC1806-shMUC16-HuR). D. However, HCC1806-shMUC16-HuR did not show significant changes in migration properties.

## Data Availability

The microarray data and materials associated with the current study are available from the corresponding author upon reasonable request.

## Abbreviations

BC: Breast cancer
FAK: Focal adhesion kinase
HuR-Hu Antigen or ELAVL1: Embryonic lethal, abnormal vision, Drosophila-like protein 1
IHC: Immunohistochemistry
IVIS: In vivo live imaging system
KD: Knockdown
KO: Knockout
MUC: Mucins
PCR: Polymerase chain reaction
RNA-IP: RNA immunoprecipitation
TCGA: The cancer genome atlas
TMA: Tissue microarray
TNBC: Triple-negative breast cancer
TPM: Transcripts per million
UNMC: University of Nebraska medical center
YBX-1: Y box protein 1
