# Supplementary figures and images for: MUC16 promotes triple-negative breast cancer lung metastasis by modulating RNA-binding protein ELAVL1/HUR
Source: Breast Cancer Res. 2023 Mar 14;25:25. doi: 10.1186/s13058-023-01630-7 (PMC10012760; doi:10.1186/s13058-023-01630-7)

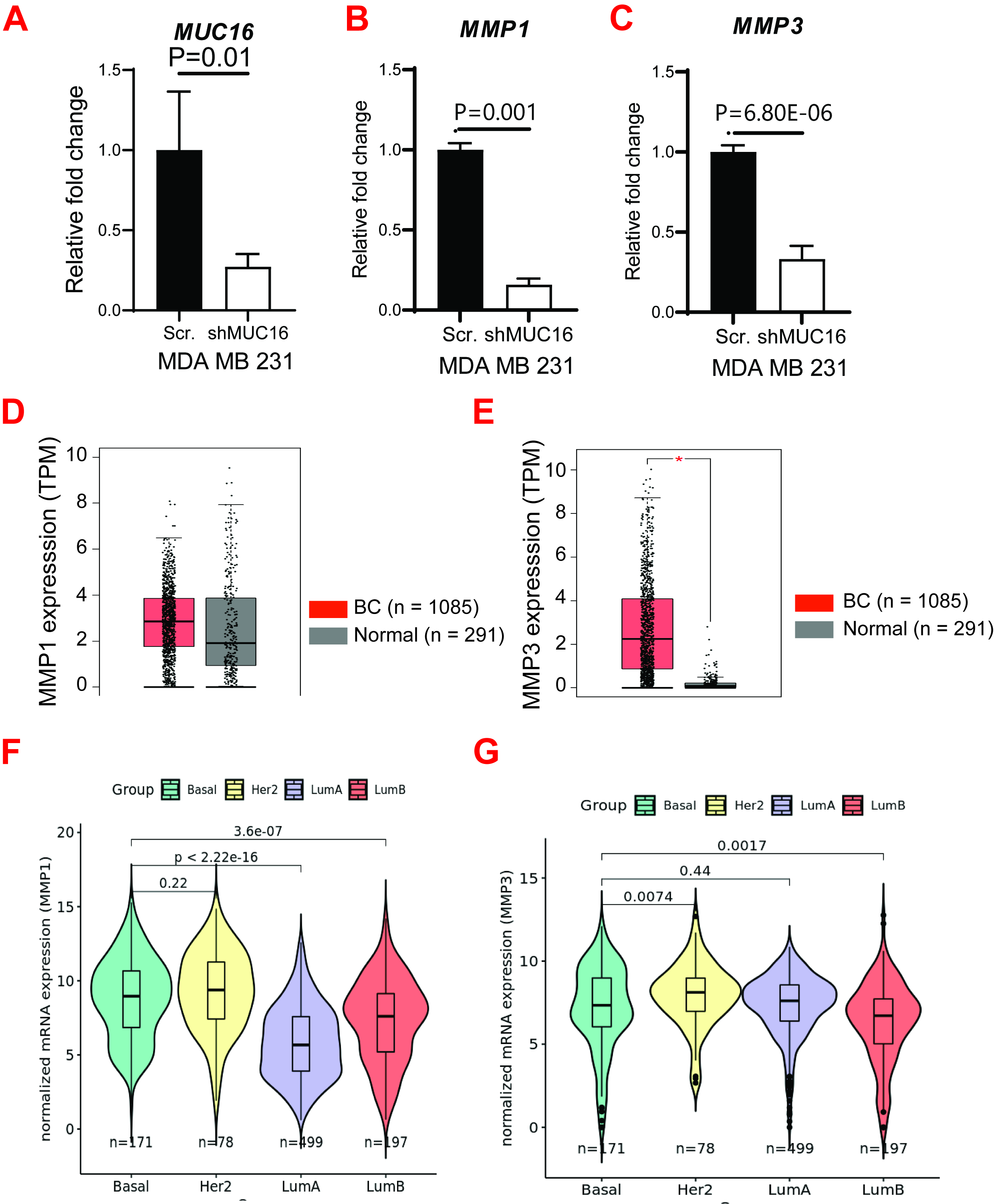

Supplement: Supplementary file 1 — Additional file 1: Fig. S1. Validation of MUC16 knockdown genes. A–C. MUC16-associated genes (MMP1 and MMP3) were validated in MDA MB 231 cells upon MUC16 knockdown. D–E. GEPIA dataset shows the expression of MMP1 and MMP3 in breast cancer patients. F, G. expression of MMP1 and MMP3 in different subtypes of breast cancer (TCGA-METABRIC). [file 13058_2023_1630_MOESM1_ESM.tif]

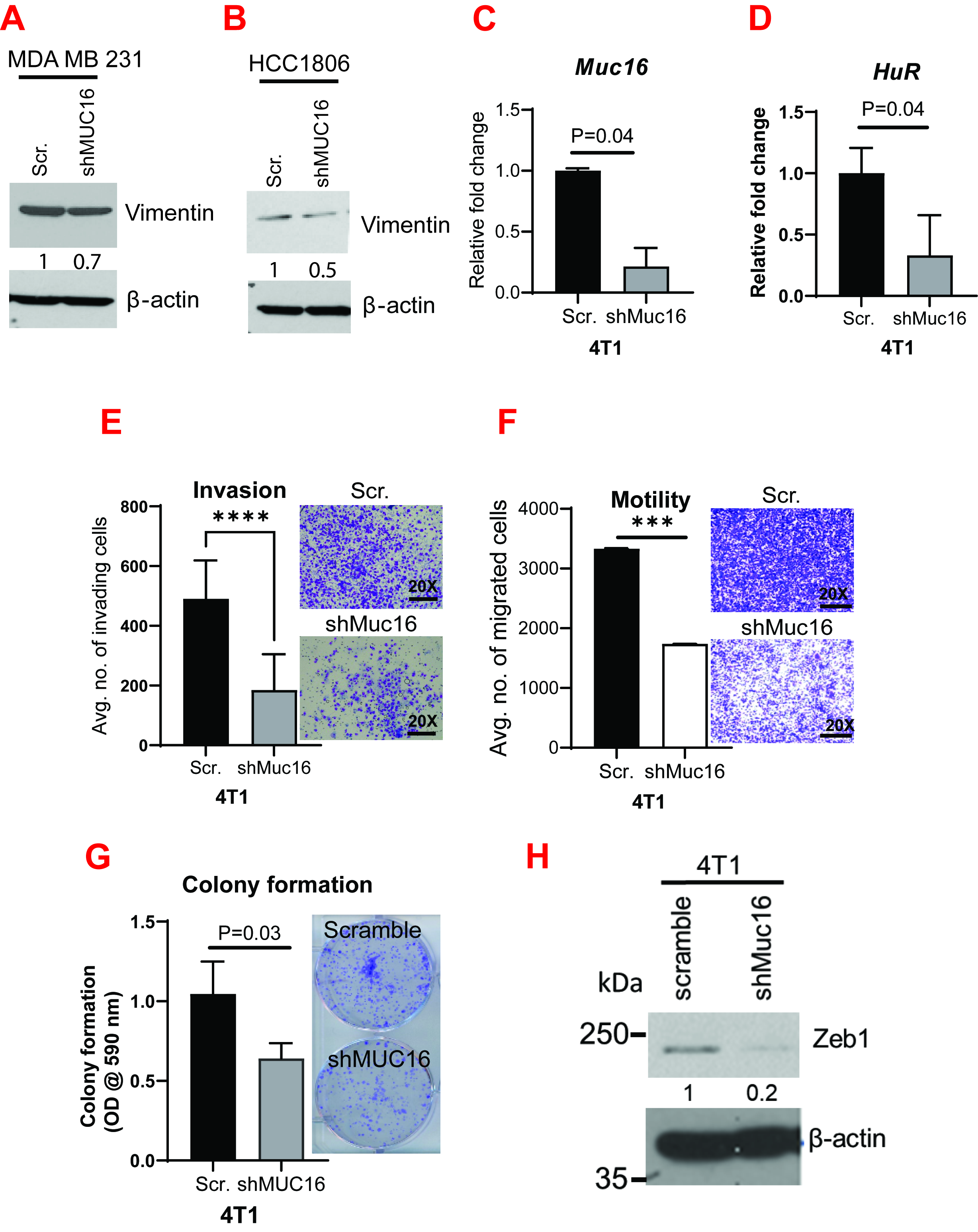

Supplement: Supplementary file 2 — Additional file 2: Fig. S2. MUC16 knockdown cells exhibit decreased metastatic markers. A–B. MUC16 knockdown (MDA MB 231-shMUC16 and HCC1806-shMUC16) shows decreased mesenchymal marker Vimentin. C–D. Bar diagram showing Muc16 knockdown and its impact on HuR expression in mouse TNBC 4T1 cells. E–G. Matrigel-coated Boyden chamber invasion, motility and colony formation assay indicating decreased invasion, migration and colony formation abilities in Muc16 knockdown in 4T1 cells. H. Decreased mesenchymal marker Zeb1 in Muc16 knockdown 4T1 cells. β-actin was used as an internal control. [file 13058_2023_1630_MOESM2_ESM.tif]

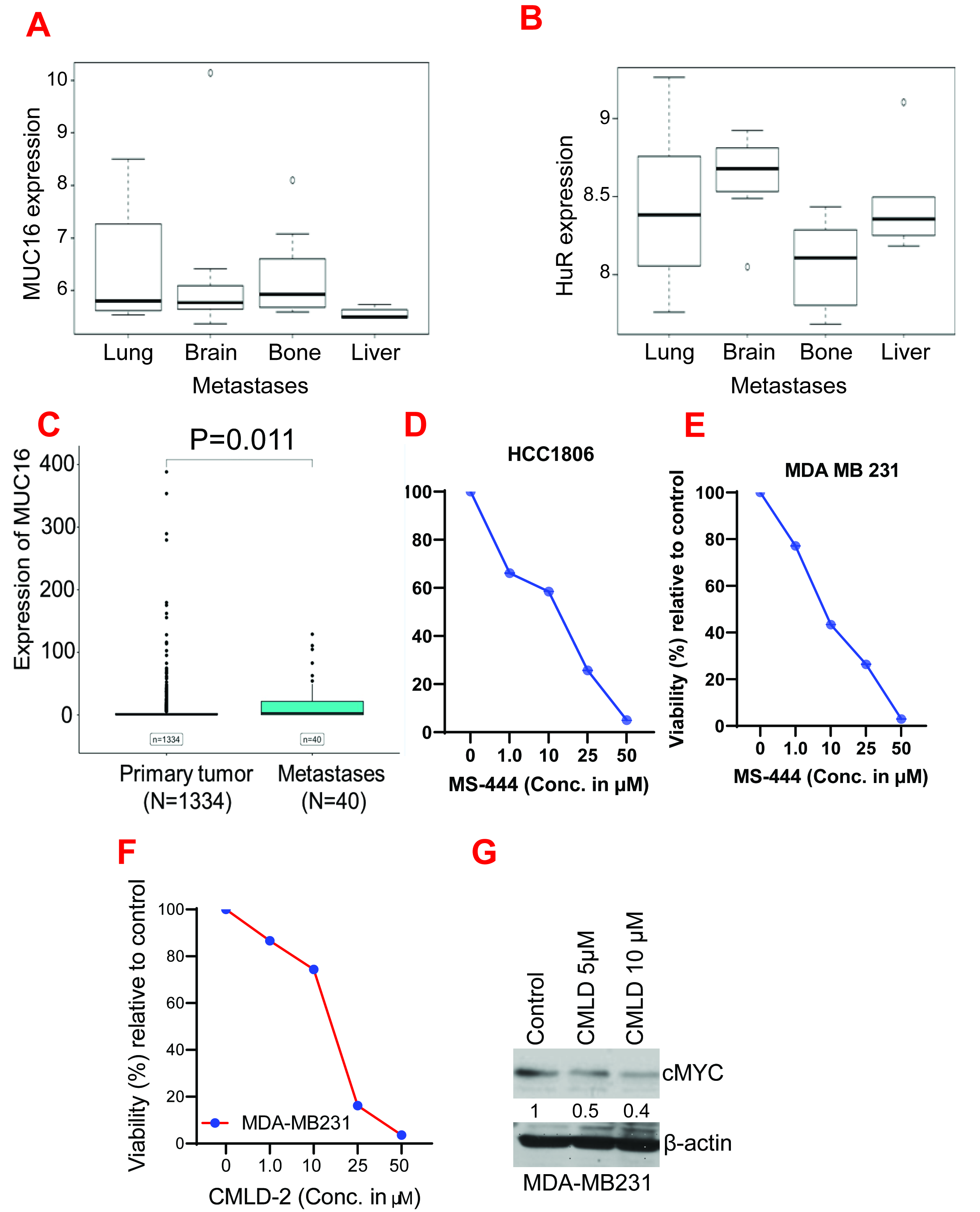

Supplement: Supplementary file 3 — Additional file 3: Fig. S3. Pharmacological inhibition of HuR and cMyc expression: A, B In silico analysis indicated the expression of MUC16 and HuR in various breast cancer metastatic tissues such as lung, brain, bone, and liver. Among all sites of breast cancer metastases, the level of MUC16 is high in lung metastases tissues. C. In silico data (GDC data portal) analysis indicated that MUC16 expression is significantly high in breast cancer metastatic tissues (individual metastasis not available). D–F. MTT assay shows the effect of MS-444 and CMLD-2 on the viability of HCC1806 and MDA MB 231 cells. (G) CMLD-2 is effectively inhibiting the cMyc expression in MDA MB 231 cells. [file 13058_2023_1630_MOESM3_ESM.tif]

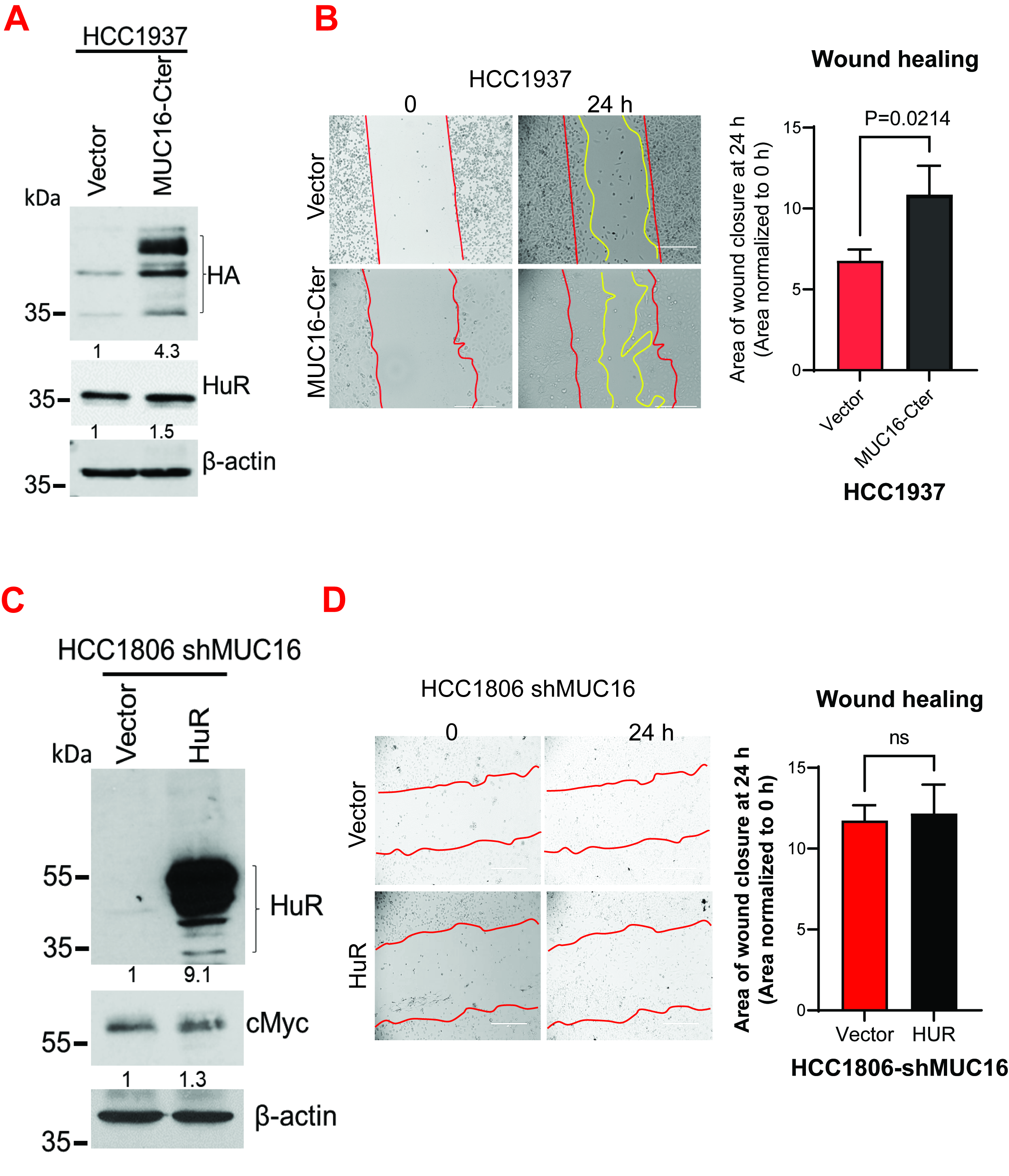

Supplement: Supplementary file 4 — Additional file 4: Fig. S4. Overexpression of MUC16-Cter and HuR in TNBC cells: A, B Ectopic expression of MUC16-Cter in TNBC cells HCC1937 and its impact of HuR expression and significantly increased wound healing properties. C. Overexpression of HuR induced cMyc expression in MUC16 silenced HCC1806 cells (HCC1806-shMUC16-HuR). D. However, HCC1806-shMUC16-HuR did not show significant changes in migration properties. [file 13058_2023_1630_MOESM4_ESM.tif]
